# Aging: An Evolutionarily Derived Condition

**DOI:** 10.3389/fgene.2012.00187

**Published:** 2012-09-27

**Authors:** Parvin Shahrestani

**Affiliations:** ^1^Department of Entomology, Cornell UniversityIthaca, NY, USA

Rose et al. (2012) communicates the importance of understanding aging in the context of Hamilton's forces of natural selection. My own experimental results, which I summarize in this article, further highlight the need for such a shift in our framework for aging research. Too often, aging is thought of as an inevitable accumulation of damage to cells, as something common to all organisms and across all adult ages, or as a physiological process. These ways of thinking about aging limit aging research. We should instead understand aging as an evolutionarily derived condition, dependent entirely on the pattern of the force of natural selection.

There is much evidence suggesting that aging is conditional on the life cycle and that the existence and non-existence of aging conforms to the expectations of evolutionary theory (Hamilton, 1966; Charlesworth, 1980, 1994; Rose, 1991). Specifically, there appears to be no aging in the absence of a steady decline in the forces of natural selection acting on mortality and reproduction. This is the case during development, when natural selection acts at full force. This does not preclude fluctuations in mortality rates during the developmental period, but it does imply the absence of a strong, persistent, and predictable deterioration in survival rates of the type seen in biological aging. Some organisms experience natural selection at full force their entire lives and are therefore expected not to age. For example, organisms with strictly symmetrical fission do not apparently exhibit aging (Bell, 1984; Martinez, 1998). In these cases, if aging were to occur, it would extinguish all the descendant lineages, wiping out any such aging species, because senescent deterioration would then accumulate from cell division to cell division. This outcome would be opposed by natural selection acting with full force, which would halt such aging among surviving species. The same is not true for asexually reproducing organisms in which reproduction is asymmetrical. These examples demonstrate that when the effectiveness of the force of natural selection is constant, aging does not occur.

In late adult life, the forces of natural selection no longer differentiate between age classes. At these late ages, there is no effective force of natural selection. This leads to a corresponding absence of consistent changes in fecundity and mortality. One prediction of the evolutionary theories is that other fitness characters, such as male virility, should also stabilize in late life. Following the virility of 1000 individual male *D. melanogaster*, I found that, as expected, male virility also reached a plateau in late life (Shahrestani et al., 2012b). This result conforms to the predictions of the evolutionary theories of late life. I also found evidence against a lifelong heterogeneity explanation for these late life virility plateaus (Shahrestani et al., 2012b).

Late life is therefore a period in which mortality, fecundity, and virility all plateau. This raises an obvious question about what happens to the constituent physiological mechanism of individuals as they transition from a period of deteriorating fitness characters to a period of stable fitness characters. In a large-scale study of more than 57,000 *D. melanogaster* from six replicate populations, I looked for changes in the patterns of physiological deterioration in the transition from aging to late life (Shahrestani et al., 2012a). Every 2–3 days throughout lifespan, I tested sample *D. melanogaster* for resistance to desiccation stress, time spent in spontaneous motion, and negative geotactic ability. At the same time, I collected mortality data, which I used to estimate the age of onset of the mortality rate plateau, which is relatively early in adult life in these populations (Shahrestani et al., 2012a). I was thereby able to dichotomize adult life in these cohorts into periods of aging and late life and to compare physiology during aging (when mortality rates increased exponentially) to physiology in late life (when mortality rates plateaued).

I found that as the cohorts demographically transitioned from aging to late life, time in motion and desiccation resistance approached stabilization, much like fitness characters do. But counterintuitively, negative geotaxis declined at a much faster rate in late life compared to its rate of decline during aging. These results suggest that late life physiology is distinct from that of aging, and that the absence of change in the effective forces of selection in late life, leads to paradoxical transitions in physiology as cohorts enter late life. From these results, I infer that the periods of aging and late life are different physiologically as a result of the very different ways in which they experience selective forces.

If late life is indeed functionally different from aging, and if this difference is due to the pattern of the forces of natural selection, then the age at which functional characters transition should evolve according to the last age of reproduction and/or survival in a population's evolutionary history. I tested this hypothesis by comparing five *D. melanogaster* populations that have been selected for accelerated development and have earlier onsets of mortality and fecundity plateaus to their five corresponding control populations that have later onsets of mortality and fecundity plateaus (Rose et al., 2002; Rauser et al., 2006). Preliminary results show that, as expected from evolutionary theories of late life, populations with earlier onsets of the mortality plateau also have correspondingly earlier onsets of physiological transitions from aging to late life (unpublished results).

My research suggests that late life is governed by very different rules than aging. In late life, chronological ages are not differentiated by forces of natural selection, so we cannot make specific predictions about patterns of physiological change in this period of adult life. This is a scenario similar to what happens during development in which the force of natural selection does not differentiate between age classes and physiological characteristics vary with respect to their improvement, deterioration, or stabilization with increasing chronological age. Between development and late life is the period of aging, in which fitness characters and constituent physiological characteristics deteriorate as a result of declining forces of natural selection.

Understanding aging in terms of a detuning of adaptation has various advantages. In principle, what can be produced by forces of natural selection can be manipulated with the use of medications or lifestyle choices. It may also be possible to alter the age of onset of the mortality plateau, leading to earlier ages for the cessation of aging. It is time to revisit older frameworks for thinking about aging, and think instead of aging as a consequence of a fall in the forces of natural selection.

## References

[B1] Bell G. (1984). Evolutionary and non-evolutionary theories of senescence. Am. Nat. 124, 600–603 10.1086/284300

[B2] Charlesworth B. (1980). Evolution in Age-Structured Populations. London: Cambridge University Press

[B3] Charlesworth B. (1994). Evolution in Age-Structured Populations, 2nd Edn London: Cambridge University Press

[B4] Hamilton W. D. (1966). The moulding of senescence by natural selection. J. Theor. Biol. 12, 12–45 10.1016/0022-5193(66)90184-6 6015424

[B5] Martinez D. E. (1998). Mortality patterns suggest lack of senescence in hydra. Exp. Gerontol. 33, 217–225 10.1016/S0531-5565(97)00113-7 9615920

[B6] Rauser C. L. Tierney J. J. Gunion S. M. Covarrubias G. M. Mueller L. D. Rose M. R. (2006). Evolution of late-life fecundity in *Drosophila melanogaster*. J. Evol. Biol. 19, 289–301 10.1111/j.1420-9101.2005.00966.x 16405599

[B7] Rose M. R. (1991). Evolutionary Biology of Aging. New York: Oxford University Press

[B8] Rose M. R. Drapeau M. D. Yazdi P. G. Shah K. H. Moise D. B. Thakar R. R. Rauser C. L. Mueller L. D. (2002). Evolution of late-life mortality in *Drosophila melanogaster*. Evolution 56, 1982–1991 10.1111/j.0014-3820.2002.tb00124.x 12449485

[B9] Rose M. R. Flatt T. Graves J. L. Greer L. F. Martinez D. E. Matos M. Mueller L. D. Shmookler Reis R. J. Shahrestani P. (2012). What is aging? Front. Gene. 3:134 10.3389/fgene.2012.00134 PMC3400891 22833755

[B10] Shahrestani P. Quach J. Mueller L. D. Rose M. R. (2012a). Paradoxical physiological transitions from aging to late life in *Drosophila*. Rejuvenation Res. 15, 49–58 10.1089/rej.2011.1201 22233126 PMC3283442

[B11] Shahrestani P. Tran X. Mueller L. D. (2012b). Patterns of male fitness conform to predictions of evolutionary models of late life. J. Evol. Biol. 25, 1060–1065 10.1111/j.1420-9101.2012.02492.x 22487207

